# Basic Helix-Loop-Helix Transcription Factor TCF21 Is a Downstream Target of the Male Sex Determining Gene SRY

**DOI:** 10.1371/journal.pone.0019935

**Published:** 2011-05-17

**Authors:** Ramji K. Bhandari, Ingrid Sadler-Riggleman, Tracy M. Clement, Michael K. Skinner

**Affiliations:** Center for Reproductive Biology, School of Biological Sciences, Washington State University, Pullman, Washington, United States of America; University of Birmingham, United Kingdom

## Abstract

The cascade of molecular events involved in mammalian sex determination has been shown to involve the SRY gene, but specific downstream events have eluded researchers for decades. The current study identifies one of the first direct downstream targets of the male sex determining factor SRY as the basic-helix-loop-helix (bHLH) transcription factor TCF21. SRY was found to bind to the *Tcf21* promoter and activate gene expression. Mutagenesis of SRY/SOX9 response elements in the *Tcf21* promoter eliminated the actions of SRY. SRY was found to directly associate with the *Tcf21* promoter SRY/SOX9 response elements *in vivo* during fetal rat testis development. TCF21 was found to promote an *in vitro* sex reversal of embryonic ovarian cells to induce precursor Sertoli cell differentiation. TCF21 and SRY had similar effects on the *in vitro* sex reversal gonadal cell transcriptomes. Therefore, SRY acts directly on the *Tcf21* promoter to in part initiate a cascade of events associated with Sertoli cell differentiation and embryonic testis development.

## Introduction

Mammalian embryos have bipotential gonads which can develop into either a testis or an ovary depending on paternal and maternal chromosomal contributions. Paternal transmission of a Y chromosome triggers testicular differentiation, whereas the contribution of paternal X chromosome results in ovarian differentiation. The bipotential gonad contains a pool of undifferentiated somatic cell precursors that during development differentiate into either testicular (Sertoli, Leydig, and peritubular myoid) or ovarian (theca and granulosa) somatic cells in response to extrinsic and intrinsic factors. The gene for the transcription factor *Sry* is located on the Y chromosome and is sufficient to induce testis fate in the bipotential gonad [1], [2], [3]. The nuclear orphan receptor steroidogeneic factor 1 SF1 and SRY cooperatively upregulate an immediate downstream gene *Sox9* through an interaction on its testis-specific enhancer [4]. Subsequently, SOX9 regulates the differentiation of precursor cells into the Sertoli cells. SOX9 expressed by Sertoli cells cooperates with FGF9 and prostaglandins [5], [6] to promote testis development. Somatic cells undergo dramatic molecular interactions during gonadal sex differentiation to ultimately influence the differentiation of germ cells. Several sex-specific genes have been shown to interact during the differentiation of gonads. For example, SRY, SOX9, FGF9, and prostaglandins have an important role in the differentiation of Sertoli cells and testis. In contrast, RSPO1, WNT4, beta-catenin, and FOXL2 antagonize SOX9 and FGF9 to promote ovarian differentiation [4], [7], [8]. Despite extensive efforts made to uncover the molecular mechanisms underlying sex determination in mammals, the downstream target genes of SRY remain poorly understood.

The process of sex determination starts with differentiation of somatic cells. Since SRY binds to HMG box sequences on the DNA, the cell fate associated genes containing HMG box binding sites in their promoter are considered potential downstream target candidates. Numerous cell type-restricted basic helix-loop-helix (bHLH) transcription factors have been identified and shown to control cell fate specification, differentiation and morphogenesis during development [9], [10], [11]. Some bHLH genes function early in embryonic development and influence a wide variety of tissues, while others act later in development and are required in the adult cells to maintain cellular differentiation. In our previous microarray analysis, a bHLH gene *Tcf21* (also called, *bHLHa23*, *Pod1*, capsulin, epicardin) was found to be highly expressed in the gonads coinciding with the expression of SRY and SOX9 [12]. A genome wide phylogenetic classification of the entire bHLH gene family has recently developed a uniform nomenclature with *Tcf21* named as *bHLHa23* and put it in perspective to other species [11], [13]. *Tcf21* null mutants exhibit male-to-female sex reversal with some germs cells entering meiosis [14]. An analysis of human, mice and rat *Tcf21* promoters showed at least three global SRY-binding sites within a 2 kb promoter region. Therefore we hypothesize that SRY interacts with *Tcf21* to promote somatic cell differentiation during male gonadal sex determination. Observations demonstrate *Tcf21* is a downstream target gene for SRY action and promotes Sertoli cell development.

## Results

### Expression of *Tcf21* gene and localization of the TCF21 protein in the rat embryonic testis

**Figure 1:**
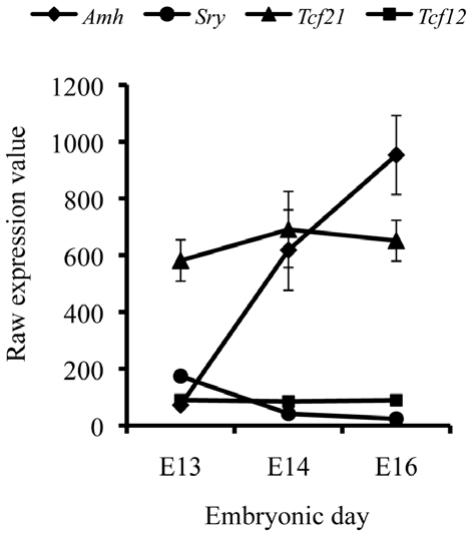
Embryonic gene expression in E13, E14 and E16 testis. Relative expression of *Tcf21*, *Sry*, *Amh*, *Tcf12* transcript levels obtained in microarray analysis in the testis of male rat at various stages of embryonic gonadal development [12].

**Figure 2:**
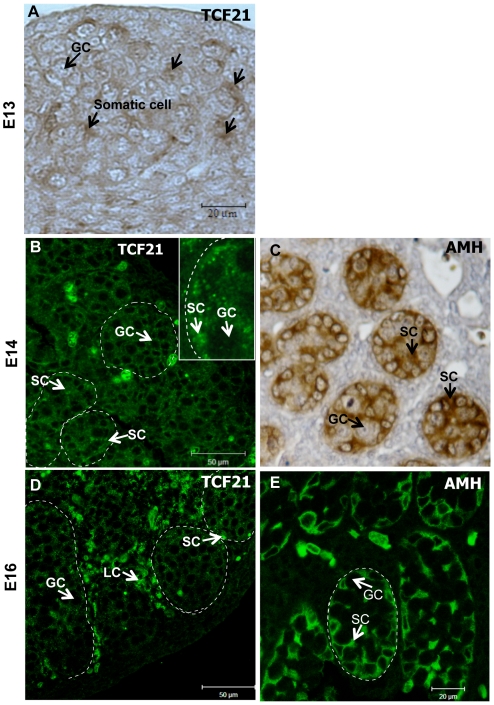
Immunolocalization of TCF21 protein in embryonic testis. (A) TCF21 protein in somatic cells of the E13 testis (arrows). (B) Localization of TCF21 protein in E14 testis. Insert shows magnified section of E14 testis. (C) AMH localization in E14 testis. AMH was stained with DAB, so brown color represents Sertoli cells. (D) TCF21 protein in E16 testis (green). (E) AMH localization in E16 testis. Abbreviations: GC = Germ Cells. SC = Sertoli Cells. LC = Interstitial cells. Dotted circles indicate testis cord structures. Data are representative of a minimum of three different experiments.

*Tcf21* expression has been detected quite early in development in the embryonic mesoderm, showing a similar pattern to *Wt1* and *Gata4* [15]. In a microarray analysis with rat gonadal RNA [12], relatively higher levels of *Tcf21* transcripts were found at the embryonic day 13 (E13) stage of testis development, after which levels are maintained during this stage of development (Fig. 1). At embryonic day 13, which is equivalent to the 13 to 18 tail somites stage, TCF21 was localized to somatic cells adjacent to germ cells (Fig. 2A). At E14, TCF21 immunopositive cells were found in the Sertoli cells and at E16 in the interstitium including Leydig cells (Fig. 2, B&D). As a positive control, AMH was used and localized to Sertoli cells (Figure 2C). The differential localization of TCF21 protein at different times of sex determination suggests differential functions at different periods of testis development. Expression patterns of TCF21 in the E13 and E14 testes indicate a transient role in Sertoli cell differentiation, whereas expression at E16 in the interstitium suggests a role during later Leydig cell differentiation [14], [16].

### Characterization of the rat *Tcf21* promoter

**Figure 3:**
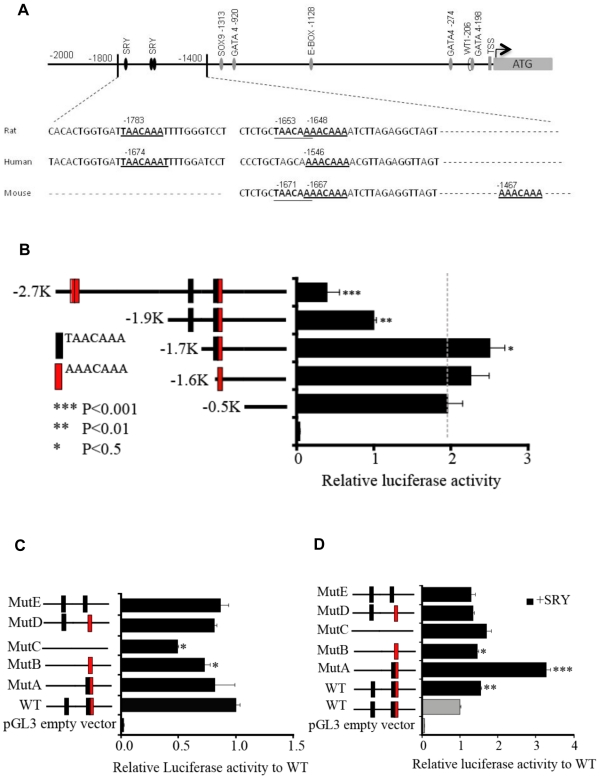
*Tcf21* promoter mutagenesis analysis. (A) Analysis of mouse, rat and human *Tcf21* promoter and SRY binding sites. Gonadal (testis) cell culture and transfection assays with relative luciferase activity compared to wild type (WT) promoter presented. Bars with asterisks are significantly different from −0.5 kb control promoter. (B) Promoter activity decreased with the length of the promoter. (C) Deletion of SRY binding motif and relative promoter activity. (D) Over-expression of SRY in E13 male primary cells increase overall luciferase activity of the mutant promoter, except for the mutant which lacks first uncoupled TAACAAA site (gray response element). Data are presented as the mean ± SEM from a minimum of three different experiments performed in replicate. The asterisks indicate (*) p<0.05, (**) p<0.01, (***) p<0.001 with a Student's t-test.

Analysis of the *Tcf21* promoter revealed three putative SRY binding sites [17] in the 2 kb upstream region from the transcription start site. A comparison was made of the genomic region of the rat *Tcf21* promoter with that of mouse and human (Fig. 3A). SRY binding sites are conserved among human, mouse and rat. There are multiple overlapping putative SRY binding sites in mouse and rat promoters. Most of the SRY binding sites are paired and separated by one or two nucleotides. In addition, the rat *Tcf21* promoter contained a binding site for SRY/SOX9 at a −1.3 k upstream region and GATA 4 in multiple locations. Multiple E-box binding sites are present within the −2 kb promoter regions.

### SRY stimulates *Tcf21* promoter activity *in vitro*

Primary cell cultures were derived from E13 testes, equivalent to 12–18 tail somite stage of male rat embryos, and used for promoter reporter assays. Binding sites for SRY and SOX9 on the *Tcf21* promoter were investigated with site specific mutagenesis for their ability to regulate the *Tcf21* promoter. E13 cultures derived from testicular primary cells or postnatal 20-day-old (P20) primary Sertoli cells were co-transfected with an *Sry* expression plasmid together with different *Tcf21* promoter fragments in a luciferase reporter system. Similar results were obtained from both E13 testis cultures or purified Sertoli cell cultures. The longer promoter constructs had lower luciferase activity (Figure 3B). Interestingly, over-expression of rat SRY (Figure 3D), mouse SRY, mouse SOX9 and human SRY (data not shown) resulted in an increased activation of the *Tcf21* promoter (Figure 3D). Point mutations of SRY motifs significantly perturbed promoter activity (Fig. 3C and D). Mutation of one of the three SRY binding sites did not result in significant change in promoter activity, while mutation of two binding sites significantly decreased the activity of the *Tcf21* promoter. Observations suggest the requirements for having at least two SRY binding sites for activating the promoter *in vitro*. Overexpression of SRY increased the activity of the *Tcf21* promoter having paired SRY binding sites (Fig. 3D).

### SRY recombinant protein binds Tcf21 promoter *in vitro*

**Figure 4:**
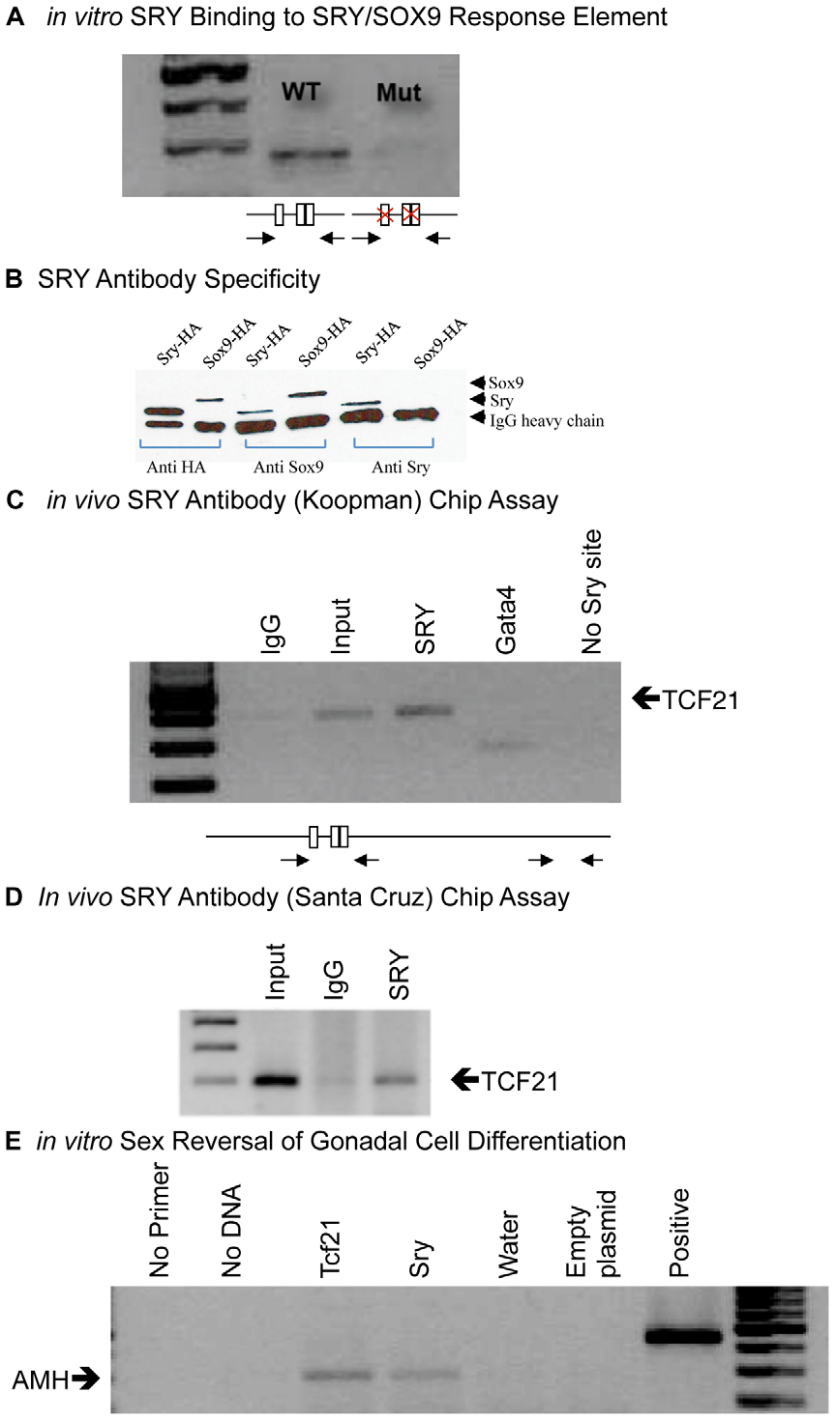
SRY *in vitro* and *in vivo* binding to *Tcf21*. (A) Immunoprecipitation-based *in vitro* pulldown assay was designed to test the physical binding of SRY protein with *Tcf21* promoter SRY/SOX9 response element oligonucleotide. Recombinant SRY-HA tagged protein was bound in vitro to 250 bp *Tcf21* promoter fragment containing three SRY binding sites. An anti-HA antibody immunoprecipitated a fragment of *Tcf21* promoter containing all three SRY-binding sites, whereas mutation of the bind site eliminated SRY binding. (B) Antibody specificity for validating *in vivo* ChIP assay. Recombinant SRY and SOX9 immunoprecipitation with anti-SRY (Santa Cruz, CA) and anti SOX9 (Koopman) antibodies followed by western blot with anti-HA antibody which was attached as a tag to those recombinant proteins. The SRY antibody specifically pulled down SRY, whereas SOX9 antibody recognized both SRY and SOX9. (C and D) *In vivo* carrier ChIP assay was performed using chromatin from the embryonic testis from E13 male embryos (14–18 tail somite stage). The anti-SRY antibody (Koopman) (C) and commercial anti-SRY antibody (Santa Cruz, CA) (D) immunoprecipitated a fragment of *Tcf21* promoter containing SRY binding sites, *in vivo*. Enriched DNA was amplified by primers flanking SRY binding site and for non-specific enrichment PCR primers were designed between −0.7 and −0.9 kb promoter which did not contain any consensus binding site for HMG box proteins (no Sry site). A control non-immune IgG (IgG) had no precipitation, while a positive control input DNA (Input) did have *Tcf21* PCR product. A positive control GATA4 antibody (GATA4) precipitation also pulled down a GATA4 site in *Tcf21* promoter. (E) Over-expression of TCF21 induced AMH expression in the E13 primary cells derived from embryonic ovaries. The TCF21 or SRY over-expression (TCF21) or (Sry) indicated, along with controls (No Primer, No DNA, Water and Empty Plasmid), with positive PCR control of L19 gene (Positive). Data is representative of a minimum of three different experiments.

The possibility that SRY binds to the defined SRY binding sites on the fragment of the *Tcf21* promoter was investigated. Reactions were set up to bind HA tagged SRY-HA recombinant protein to the *Tcf21* promoter oligonucleotides with or without mutated SRY binding sites followed by immunoprecipitation (IP) with anti-HA antibody. Enriched fragments were analyzed by PCR with primers flanking the binding sites (Fig. 4A). Affinity purified non-immune mouse IgG was used as a negative control in the experiment. Anti-HA antibody pulled down the oligonucleotide with intact SRY binding sites, but not the one with mutated SRY binding sites (Fig. 4A).

The specific SRY antibody developed by Dr. Peter Koopman's laboratory, Queensland University, Brisbane, Australia was shown to bind SRY, but not SOX9 [6]. In order to validate a commercially available anti-SRY (Santa Cruz, Santa Cruz CA) antibody for ChIP assay, *in vitro* immunoprecipitation was performed using recombinant SRY and SOX9 proteins. Enrichment of target protein was detected by western blot using antibodies against the HA tags for the recombinant proteins. The commercial SRY antibody did pull down SRY, but did not pull down SOX9 recombinant protein (Figure 4B). The SOX9 antibody recognized both SRY and SOX9 recombinant proteins (Figure 4B). Therefore, the SRY antibody was specific for SRY and thus used in the ChIP assay.

### SRY binds *in vivo* to the Tcf21 promoter during male sex determination

There has been little progress toward identifying *in vivo* downstream targets of SRY. This is in part due to technical limitations of procedures such as *in vivo* ChIP assay which traditionally requires a large amount of starting material from the embryonic testis. For example, a conventional ChIP assay requires a large amount of chromatin which is equivalent to pooled gonads of five to six hundred embryos [6], [18]. The techniques used in the present study overcome these technical difficulties. The present study used a modified *in vivo* ChIP assay with isolated testis samples from 20 to 25 testes at the 12 to 18 tail somite stage of rat embryo development. The promoters of any genes that SRY directly regulates at the time of gonadal sex determination should be pulled down from the chromatin mixture by anti-SRY antibody. To test whether SRY associates with a fragment of *Tcf21* promoter, PCR primers were designed to amplify the *Tcf21* promoter regions flanking an SRY binding site. As a negative control, primers were designed to a promoter region which did not contain any HMG box sites or putative SRY binding sites. The SRY antibody obtained from Dr. Peter Koopman, Brisbane, Australia, pulled down the fragment of *Tcf21* promoter containing the predicted SRY binding site, but not the negative control region without its binding site (Fig. 4C). As a positive control in the ChIP assay, anti GATA4 antibody was used. Three GATA4 response element sites are present at different regions of the *Tcf21* promoter and were used as a positive control. A ChIP assay for GATA4 was found to immunoprecipitate and identify the *in vivo Tcf21* promoter (Fig. 4C). The anti SRY from Dr. Peter Koopman was previously shown to be specific for mouse SRY and was found to pull down the Tcf21 promoter from developing rat E13 gonads (Fig. 4C). A commercially available anti SRY antibody (Santa Cruz, Santa Cruz, CA) was found to be specific for SRY (Fig. 4B) and also pulled down the Tcf21 promoter in the ChIP assay from developing E13 testis (Fig. 4D). Therefore, two different anti SRY antibodies in the ChIP assay demonstrated that SRY directly binds the *Tcf21* promoter during male gonadal sex determination. Positive controls such as TESCO which is considered a downstream target for SRY [4], has not been characterized in the rat, so current study did not focus on TESCO as a positive control. Instead we have initiated genome-wide ChIP-Chip analysis of SRY targets in embryonic testis during sex determination.

### TCF21 promotes *in vitro* sex reversal of embryonic ovarian cells to induce Sertoli differentiation

The above *in vitro* and *in vivo* results demonstrate that SRY interacts with the *Tcf21* promoter during male sex determination and testis differentiation. Tcf21 expression level was similar to that of *Wt1* and *Gata4* at E9.5 and E10.5 [15], whereas during male sex determination transcripts for this gene remained elevated in the rat. The homozygous knockout mutant for *Tcf21* showed abnormal gonadogenesis, lost testis cord structures, abnormal vascular innervations into the testis, and reduced Sox9 expression in the testis [14]. These results suggest that TCF21 is involved in male sex determination and testis differentiation. An embryonic E13 ovary culture system was used to test whether TCF21 can promote *in vitro* sex reversal and the induction of the expression of a male marker gene of testis differentiation. Expression plasmids for *Tcf21* or *Sry* were over-expressed in subcultures (less than 12) of primary cell cultures derived from E13 rat ovaries. The objective was to promote an *in vitro* sex reversal from an ovarian somatic cell population to a testicular somatic cell population. TCF21 over-expression induced the expression of anti Müllerian hormone (AMH) which is a specific marker of Sertoli cell differentiation in the embryonic testis, but not expressed by the embryonic ovary or ovarian culture, Figure 4E. As expected, SRY over-expression in the embryonic ovarian cells also induced AMH expression (Figure 4E). This observation suggests that TCF21 promotes embryonic Sertoli cell differentiation in a similar manner as SRY.

**Figure 5:**
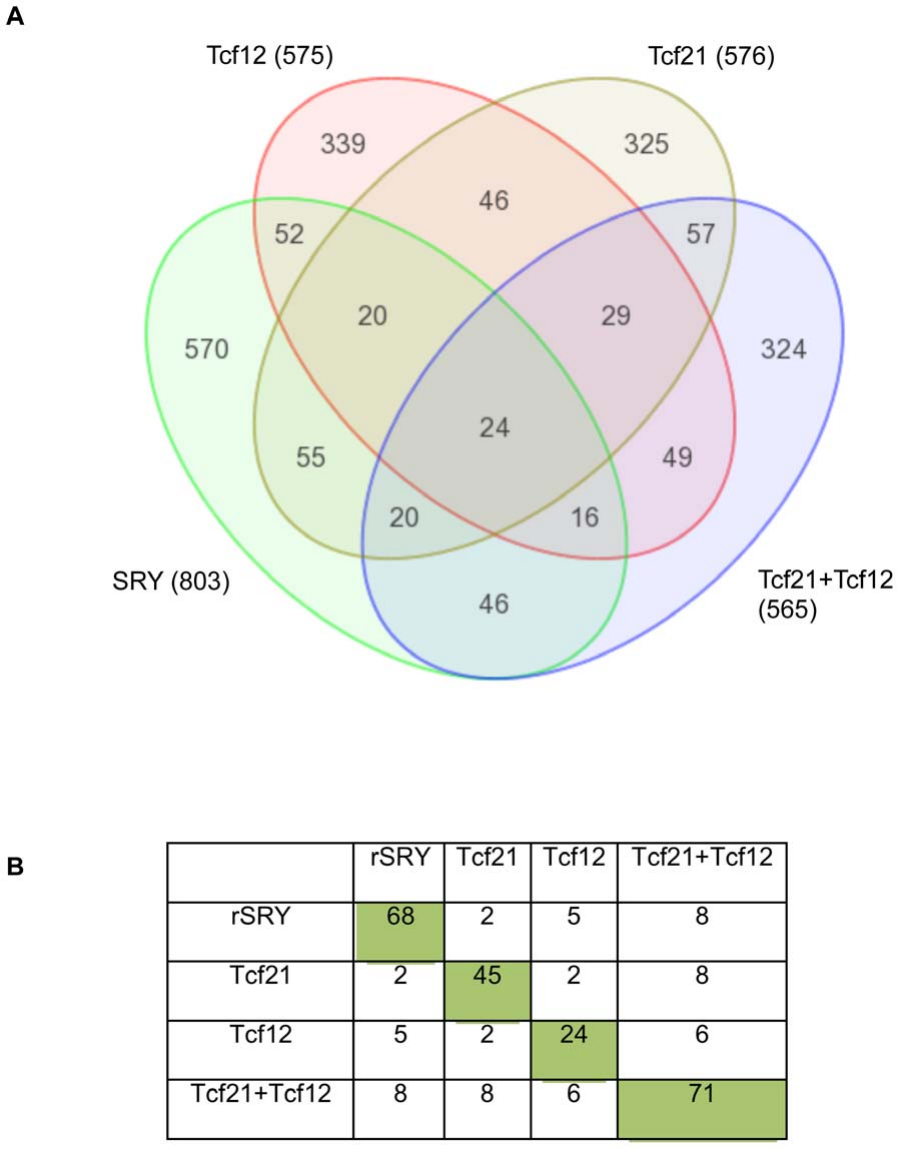
Transcriptome analysis of SRY and TCF21 actions. Venn diagram (A) with overlap of total gene sets (p<0.05) altered in response to various expression constraints (*Sry*, *Tcf21*, *Tcf21* plus *Tcf12*, *Tcf12*). Total genes in the gene sets and overlap numbers are presented. These gene sets are statistically significant increase genes, but with no further cut-off parameters. (B) Restricted gene sets overlapped in female E13 cultures involving gene sets with altered expression with >1.2 fold change, p<0.05, and mean difference >10.

The cell culture transcriptomes were examined to assess the *in vitro* sex reversal of the embryonic ovarian cell culture to a testis cell differentiated state on a genome wide level. Microarray analysis of the embryonic cell cultures were investigated in the absence or presence of SRY, TCF21 or TCF21 with its potential binding partner TCF12/REB-alpha. All bHLH transcription factors dimerize and due to the high level of *Tcf12* expression in the embryonic testis (Figure 1), the combined actions of TCF21 and TCF12 were also assessed. TCF12 is also known as REB-alpha which is somewhat ubiquitously expressed and a binding partner of numerous bHLH factors [19]. Over-expression of SRY in the embryonic ovary cell culture promoted a transcriptome more similar to the testis with approximately 800 significantly increased transcripts, Figure 5A. Interestingly, TCF21 also promoted a transcriptome with approximately 20% overlap with that induced by Sry, Figure 5A. The combined actions of TCF21 and TCF12 induced a more dramatic increase in AMH expression, data not shown, and a transcriptome with overlap with TCF21 and SRY, Figure 5. Observations support a potential role for TCF12 as a potential partner for TCF21. A list of the genes with the most highly increased expression induced by SRY and TCF21 are presented in Table S1. An extended list of the genes altered after TCF21, SRY or TCF21 and TCF12 over-expression are presented in Table S2. Observations demonstrate TCF21 and TCF12 promote some similarities in the *in vitro* sex reversal transition in the transcriptome as SRY, supporting a role of TCF21 as a downstream target of Sry during male gonadal sex determination.

## Discussion

**Figure 6:**
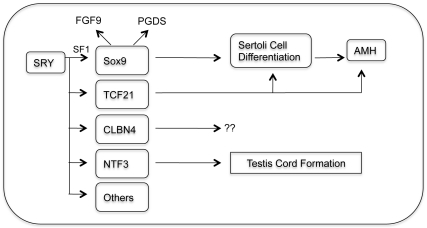
Summary of SRY downstream genes. Proposed downstream actions of SRY on *Sox9* and *Tcf21* genes, along with *Clbn4*, *Ntf3*, and others yet to be identified. TCF21 induction of Sertoli cell differentiation and expression of marker genes such as *Amh* indicated. Combined actions of SRY and SF1 on *Sox9* expression and actions on *Fgf9* and *Pgds* expression indicated.

Since the discovery of SRY as a master male sex determining factor several efforts have been made to identify its downstream target genes. Whether *Sox9* is the only downstream gene that carries out all functions necessary for initiating male sex development or whether there are multiple SRY target genes that cooperatively act to induce testis-specific somatic cell differentiation remains to be determined. Recent developments suggest that SRY and SF1 cooperatively activate *Sox9* expression through interactions with its enhancer region, confirming *Sox9* as one of the SRY target genes [4] (Figure 6). The SRY protein has been shown to interact *in vivo* with the promoter enhancer region of the Cerebellin 4 precursor gene (*Cbln4*), which is present in Sertoli cells during early embryonic development [18] (Figure 6). Although, the exact function of *Cbln4* gene in testis differentiation is not clearly understood yet, this gene has been suggested as one of the downstream target genes of SRY. Using a similar approach to that taken in the current study we have found SRY directly interacts and regulates the promoter for neurotrophin 3 (*Ntf3*) [20], that has previously been suggested to have a critical role in testis cord formation [21], [22] (Figure 6). A major hindrance in finding SRY target genes is the technical limitation to *in vivo* chromatin immunoprecipitation (ChIP) methods. Using an *in vivo* carrier ChIP method the current study demonstrates that SRY interacts with the bHLH family transcription factor *Tcf21* during male sex determination. Observations provide insights into the mechanism of SRY regulation of Sertoli cell differentiation and the factors regulating (e.g. TCF21) cell differentiation during male gonadal sex determination.

Numerous cell type-restricted basic helix-loop-helix (bHLH) transcription factors have been identified and shown to control cell fate specification, differentiation and morphogenesis during embryonic development, while others act later in development and are required in the adult cells to maintain cellular differentiation. Examples include MESP in heart cell differentiation, MYOD in muscle differentiation, and Neurogenin in neuron differentiation [23], [24], [25]. In an embryonic rat and mouse testis microarray *Tcf21* was found as one of the most highly expressed transcription factor genes [12], Figure 1. The presence of TCF21 protein in the somatic cells of the embryonic testis was confirmed immunohistochemically. Nuclear localization of TCF21 was observed in the Sertoli cell in testis of the E13 and E14 testis, whereas it appeared in the interstitial cells in E16 testis suggesting it has multiple functions during testis differentiation. Prior to male sex determination *Tcf21* mRNA is localized in the gonadal ridges and its expression pattern was found to be similar to *Wt1* and *Gata4* [15]. Gene knockout studies suggested that *Tcf21* null mutant male mice were partially sex-reversed [14]. Combined observations suggest *Tcf21* has multiple roles in male sex determination and testis morphogenesis.

Most of the genes that are highly expressed during the period of male sex determination have SRY or SOX9 binding sites in their promoters [17]. Since the *Tcf21* promoter harbors three SRY binding sites in the −2 Kb promoter region, it was hypothesized that SRY may bind these response elements and regulate the activity of the core promoter. Over-expression of SRY in the primary embryonic testis cell culture increased the *Tcf21* promoter activity. This *Tcf21* promoter activity was significantly reduced when all three SRY binding sites were mutated. SRY seemed to be able to activate the *Tcf21* promoter if any two of the three binding sites were intact. An oligonucleotide pull-down assay was used to confirm SRY directly binds to the *Tcf21* promoter SRY binding sites (Figure 4A). Although these *in vitro* promoter actions and binding studies support a potential role for SRY regulation of *Tcf21*, SOX9 can have similar activities and a direct role of SRY is uncertain *in vivo*. An example of a gene that has similar *in vitro* observations, but was found *in vivo* to primarily respond to SOX9 is prostaglandin synthase (PGDS) [6] (Figure 6). To investigate the potential direct SRY binding to the *Tcf21* promoter *in vivo* a ChIP assay with chromatin samples from embryonic testis undergoing sex determination and cellular differentiation was performed. The major limitation in running ChIP assays with embryonic gonad samples is the amount of chromatin required. This limitation has hindered efforts made to find downstream target genes of SRY. The current study adopted the ChIP protocol developed by O'Neill et al (2006) [26] and modified to meet conditions with rat SRY protein. Observations demonstrate a ChIP with SRY antibody immunoprecipitated from embryonic testes the *Tcf21* gene promoter. *Tcf21* appears to be one of the downstream target genes of SRY. To identify other SRY target genes an SRY ChIP is currently being subjected to ChIP-Chip whole genome promoter tiling arrays.

*Tcf21* null mutants have several testis phenotypes [14] suggesting multiple roles for TCF21 in testis organogenesis. *Tcf21* was over-expressed in primary embryonic ovarian cells derived from 15 tail somite stage female embryos (E13) to investigate the actions of TCF21. *Tcf21* over-expression induced *Amh* expression in the female cells and so provided evidence of its role in the induction of testis somatic cell differentiation (Figure 4E). *Amh* is not expressed in the ovary at this time of development, but Sertoli cells in the embryonic testis do express *Amh*. In contrast, *Tcf21* null mutants did not completely lack SOX9 and AMH expression because the loss of TCF21 was likely compensated for by yet undefined tissue specific binding partners. This phenomenon has been seen for heart muscle differentiation in *Mesp1* mutants [23], [27] and prostate differentiation in *NCoA1* mutants [28]. The over-expression of *Tcf21* in the fetal ovarian cell culture promoted an SRY-like transcriptome and cellular differentiation similar to normal testis development. SRY and TCF21 both induced similarities in the transcriptomes and *in vitro* gonadal sex reversal in the ovarian cell culture. The group of specific genes identified will be useful to consider in further elucidation of the cascade of gene expression in testis differentiation. In addition to the identification of the *in vitro* and *in vivo* association of SRY with the *Tcf21* promoter, a functional role of TCF21 in promoting *in vitro* sex reversal and a SRY-like gene expression profile was observed.

A distinct function of TCF21 in testis differentiation is associated with fetal Leydig cell differentiation. Excessive proliferation of P450SCC and SF1 expressing cells were observed in the gonads of *Tcf21* null mice [29]. TCF21 expression allows fetal Leydig cells to maintain stem cell like characteristics. These results can be explained by observations that TCF21 suppresses SF1, NOTCH1 and hedgehog signaling which are required for differentiation of fetal Leydig cells [16], [30]. In the rat we observed differential immunolocalization of TCF21 during male sex determination and testis differentiation. At the onset of sex determination when SRY is critical for the induction of Sertoli cell fate determination, TCF21 is primarily localized to Sertoli cells during testis cord formation (E13–E14). Later in testis differentiation as the interstitial cells and Leydig cell differentiation is initiated the TCF21 is primarily localized in Leydig cells (E16). Therefore, TCF21 has a transient role in the initial differentiation of Sertoli cells in response to SRY, and then a later role in the initiation of Leydig cell differentiation. The hypothesis is that TCF21 likely promotes a cascade of other factors, such as bHLH proteins, to continue the subsequent differentiation.

Combined *in vitro* and *in vivo* observations have demonstrated that TCF21 is a downstream target of SRY and plays an important role in testis differentiation (Figure 6). Our results also provide insights into the possibility for bHLH protein involvement in Sertoli and Leydig cell differentiation. Since bHLH transcription factors have a critical role in the differentiation of many tissues and cell types, a role in mammalian sex determination and gonadal cell differentiation will be an interesting future area of research. Experiments are in progress to determine the cascade of genes following TCF21 and SRY expression and their roles in testis differentiation and male sex determination.

## Materials and Methods

### Tissue preparation and Histology

Harlan Sprague-Dawley rats were kept in a temperature controlled environment and given food and water *ad libitum*. Estrous cycles of female rats were monitored by cellular morphology from vaginal smears [31]. Rats in early estrus were paired with males overnight and mating confirmed by sperm positive smears, denoted day 0 of pregnancy. Pregnant rats were euthanized at E13, E14, and E16 of pregnancy, and embryonic gonads were collected for histological analysis. Sex was determined by PCR using primers specific for *Sry* on genomic DNA isolated from embryo tails as previously described [32]. All procedures were approved by the Washington State University Animal Care and Use Committee (IACUC approval # 02568-018). Tissue specimens were fixed in Bouin's solution for 1 h and embedded in paraffin using standard procedures. Serial sections of 4 µm were stained with hematoxylin and eosin (H&E) using standard procedures [33].

### Immunohistochemistry

Embryonic testis sections [34] were deparaffinized, rehydrated through a graded ethanol series, boiled 10 minutes in 10 mM sodium citrate buffer to expose the antigens, washed with 0.1% Triton-X solution, and then blocked with 10% serum of the species secondary antibody was raised in for 30 min prior to incubation with 0.5 µg/ml primary rabbit anti-TCF21 antibody for 18 h (ABcam). The sections were then washed with PBS and incubated with 1∶3000 diluted Alexa Fluor 488 labeled secondary antibody for 45 min (goat anti-rabbit IgG; Invitrogen, Eugene, OR). Slides were mounted with Vecta-Shield plus DAPI (Vector Laboratories Inc.), sealed with coverslips, and analyzed using fluorescence confocal microscopy (Zeiss). Negative control experiments were performed using a non-immune IgG at 0.5 µg/ml (rabbit IgG; Sigma, *St. Louis, Mo*). AMH localization was performed using 5 µg/ml primary anti-MIS antibody for 18 h (R&D Systems, Minneapolis, MN) and 1∶3000 diluted Alexa Fluor 488 labeled secondary antibody (donkey anti-goat IgG; Invitrogen, Eugene, OR) using the protocol above. Non-fluorescent staining was performed using DAB staining method.

### Reporter Plasmid Preparation

All the primers used in plasmid preparation are listed in the Table S3. In order to prepare *Tcf21* promoter/reporter vectors, different size fragments (−2.7 k, −1.9 k, −1.7 k, −1.6 k, and −0.5 k) of the rat genomic DNA upstream of the coding region was amplified (for PCR primers, see Table S3) and cloned into the pGL3-Basic luciferase reporter vector (Promega, Madison, WI) using the SmaI and KpnI sites of the multiple cloning region [35]. To generate mutant promoter/reporter constructs, the SRY binding consensus sites in the promoter were mutated using complementary oligos (Table S3). Mutagenesis was performed using i-proof Taq polymerase (BioRad) according to the manufacturer's directions. All constructed promoter vectors were sequence verified. Mouse *Sry* and *Sox9* expression vectors were generously gifted by Dr. Peter Koopman (University of Queensland, Australia) to M.K.S. Rat Sry expression construct was prepared as described [20]. Briefly, a full length rat Sry expression plasmid with a MYC tag was produced by amplifying the single exon from rat genomic DNA and cloned into pCMV-MYC expression vectors (Clontech, Mountain View, CA) using the BglII and NotI restriction sites. In order to prepare recombinant rat SRY protein, Myc-tagged rat SRY coding region was inserted into pcDNA 3.1 vector and *in vitro* transcribed & translated using a TnT T7 quick *in vitro* Transcription and Translation kit (Promega).

### Cell Preparation and Culture

#### Sertoli cell culture

All cell preparation and transfections were performed according to the protocol developed in the laboratory as previously described [36]. All animal procedures and protocols were approved by the Washington State University Animal Care and Use Committee. Decapsulated testes were minced with razor blades. Fragments were then digested with trypsin (1.5 mg/ml, Life Technologies, Gaithersburg, MD) to remove the interstitial cells followed by collagenase (1 mg/ml type I, Sigma) for removal of peritubular cells and then hyaluronidase (1 mg/ml, Sigma) for removal of germ cells. Sertoli cells were plated under serum free conditions in 24-well Tissue Culture Plates (Falcon Plastics, Oxnard, CA) at 1×10^6^ cells/well. Cells were maintained in 5% CO2 atmosphere in Ham's F-12 medium (HyClone) at 32°C [37].

#### E13 testis cell culture

E13 embryos (12 to 18 tail somite-stage) were collected from timed-pregnant females as described above [34], [38]. Gonads from E13 animals were dissected and each pair of gonads from individual animals was placed into one well of a 24 well plate with 500 µl Ham F-12 medium until embryos could be sexed as described above. The male gonads were then pooled and digested with trypsin (2.5%) and collagenase (1 mg/ml type I) plus DNase (3 mg/ml) to disassociate the cells. All the cells from the digested testes were then cultured on 100 mm plates in Ham's F-12 with 10% bovine calf serum (Sigma). Cells initially multiplied well in culture and were split two times (1∶2) as they reached confluence, at which point cell division slowed considerably. Cells were maintained in culture, changing medium every three days, until growth plaques were observed at approximately one month. These growth plaques were then collected for further propagation and frozen stocks were prepared for subsequent cell splitting such that cells could be maintained at fewer than 12 subcultures. E13 female primary cells were also prepared accordingly using female gonads from E13 (14 to 18 tail somite-stage female embryos).

### Transfection Procedures

#### Sertoli cell transfection

Sertoli cells cultured for 48 hours were transfected by the calcium phosphate method coupled with hyper osmotic shock (10% glycerol) as previously described [35], [36]. Briefly, 2 µg promoter reporter plasmid with or without 1 µg expression plasmid in 150 µl transfection buffer (250 mM CaCl2 mixed 1∶1 (v∶v) with 2× HEBES (28 mM NaCl, 50 mM HEPES, and 1.47 mM Na2HPO4 at pH 7.05)) was added to each well of a 24 well plate containing 1×10^6^ Sertoli cells in 1 ml Ham's F-12 medium and incubated at 32°C for 3.5 hours. Following incubation, the cells were subjected to hyper-osmotic shock. The medium was aspirated and 1 ml of 10% glycerol in Hanks' Balanced Salt Solution (HBSS, Invitrogen) was added for 3 minutes. Wells were washed twice in HBSS before fresh Ham's F-12 with 0.01% BSA was added. Following a 72 hour incubation cells were harvested for luciferase assays. Medium was aspirated and 100 µl of 1× cell lysis solution (Promega) was added per well. Plates were frozen and thawed before cell lysate was collected. For detection of luciferase reporter activity 20 µl of Sertoli cell lysate was mixed with 100 µl of luciferase substrate (Promega) and luciferase activity detected on a Wallac Victor II 1420 instrument.

#### E13 cultured testis cell transfection

Cells between sub 8 and 12 were transfected using Lipofectamine 2000 (Invitrogen, Carlsbad, CA) [39]. Two µg promoter reporter plasmid with or without 0.5 µg expression plasmid was mixed with 2 µl Lipofectamine 2000 in 100 µl Opti-MEM medium (Invitrogen) for each well of a 24 well plate. This 100 µl mix was added to each well containing ∼90% confluent cultured E13 testis cells in 1 ml Ham's F-12 medium without antibiotics and incubated at 32°C for 24 hours. After 24 hours medium was aspirated from cells and replaced with 1 ml Ham's F-12 with 10% serum. Cells were incubated 72 hours and collected for luciferase assays as described above for Sertoli cells. For over-expression, cells were transfected in 6 well plates with 4 µg expression construct for TCF21-pCMV-myc, rat SRY-pCMV-myc and rat TCF12-pCMV-HA. Cells were collected after 6 days and RNA was extracted using Trizol. RNA was reverse transcribed and cDNA was used for PCR. At least three over-expression experiments were made with both E13 male and female cell cultures.

### Immunoprecipitation Pull-Down Assay

SRY recombinant protein with HA tag was synthesized *in vitro* by using *Sry* expression construct and an *in vitro* transcription and translation kit according to the manufacturer's instruction (Promega) [40]. Oligonucleotides corresponding to *Tcf21* promoter with or without SRY binding site were generated by PCR amplification with primers listed in the Table S3. DNA-protein binding was performed in 30 ul reaction mixture containing 100 mM KCl, 1 mM MgCl_2_, 10 µM ZnSO_4_, 10 mM Tris, pH 7.5, 4% glycerol, 0.1% Triton X-100, 1 mg/mL BSA, 1 µg of poly(dIdC)/poly(dAdT), 0.5 mM DTT and protease inhibitor cocktail mini tabs (Sigma). Binding reaction continued for an hour at room temperature. Protein-A Sepharose beads were preswollen in the cChIP incubation buffer (50 mM Nacl, 20 mM Tris HCl pH 7.5, 20 mM Na-butyrate, and 5 mM EDTA) overnight. Before use, beads were incubated with anti-HA antibody or with non-immune IgG as a negative control. Following gentle centrifugation (600× g) for 5 minutes, beads were treated with protein-oligo mixture and incubated with gentle rotation overnight at 4 degree Celsius. In this way the antibodies, while bound to the beads, would attach to their matching proteins and capture the oligo fragments specifically bound to the proteins. Beads were washed three times with wash buffer containing 50 mM, 100 mM and 150 mM NaCl. Pulled-down oligos were eluted in a buffer containing 0.5% SDS and purified in PCR purification columns according to manufacturer's instruction (Promega). Purified oligo DNA was subjected to PCR with primers designed to flank that particular sequence of *Tcf21* promoter.

### Chromatin Immunoprecipitation (ChIP) Assay

#### *In vivo*

Carrier ChIP (cChIP) assay was adopted from O'Neill et al., (2006) [26] and conditions were modified to meet the condition for immunoprecipitating SRY. To run ChIP assay, male and female gonads (gonad+mesonephros) were dissected from approximately twenty to twenty five 13 dpc (12–18 tail somite stage) rat embryos per array and snap-frozen in liquid nitrogen. *Drosophila* SL2 cells were used as a carrier. Densely-grown cells (approximately 5×10^7^ cells) were pelleted and washed three times in ice-cold PBS, 5 mM sodium butyrate and resuspended in 0.5 ml NB buffer (15 mM Tris-HCL, pH 7.4, 60 mM KCl, 15 mM NaCl, 5 mM MgCl_2_, 0.1 mM EGTA, 0.5 mM 2-mercaptoethanol, 0.1 mM PMSF). Frozen testis samples were thawed, mixed with SL2 cells and homogenized to make single cell suspension. Nuclei were pelleted, resuspended in 20 ml NB buffer, 5% (vol/vol) sucrose, pelleted and resuspended again in 5 ml digestion buffer (50 mM Tris-HCl pH 7.4, 0.32 M sucrose, 4 mM MgCl_2_, 1 mM CaCl_2_, 0.1 mM PMSF). Following micrococcal nuclease digestion (NEB, USA) for 10 minutes at 37 degree, the digested samples were gently spun (800×g) for 10 minutes and supernatant set aside on ice. The pellet was resuspended in 250 ul incubation buffer (50 mM Nacl, 20 mM Tris HCl pH 7.5, 20 mM Na-butyrate, 5 mM Na2EDTA, and 0.1 mM PMSF) and again centrifuged gently for 10 minutes. Both the supernatants were pooled and a fraction (50 ul) out of it was kept aside to use as input. The remaining supernatant was incubated with either non-immune IgG or anti-SRY or anti-Gata4 antibodies at 4°C overnight. After incubation with 100 µl of preswollen protein A-Sepharose beads (Sigma) for 2 h at 4°C, the bead-bound immunoprecipitates were centrifuged gently and washed five times with wash buffer (50 mM TrisHCl pH 7.5, 10 mM EDTA, 5 mM Na butyrate and 50–150 mM NaCl). The protein-DNA complexes were incubated at room temperature with elution buffer (1% SDS in TE) and centrifuged at 11500×g at 4°C for 10 minutes. Elution was repeated two times and eluted DNA was pooled. Co-immunoprecipitated DNA was purified by proteinase K digestion, phenol/chloroform extraction, and ethanol precipitation. Final concentration of immunoprecipitated DNA varied from 100 to 300 ng per assay. Two rounds of PCR reactions were run for 25 cycles before evaluating samples by gel electrophoresis. Identity of the PCR-amplified fragments was verified by sequencing. Primers used to investigate the enrichment of *Tcf21* promoter fragments by PCR are listed in Table S3.

### Microarray Analysis

mRNA processing and hybridization were performed at Genomics Core Laboratory, Center for Reproductive Biology, Washington State University, Pullman, WA using standard Affymetrix reagents and protocol [41]. Briefly, mRNA was transcribed into cDNA with random primers, from the later, cRNA was transcribed, and from that, single-stranded sense DNA was synthesized which was fragmented and labeled with biotin. Biotin-labeled fragmented ssDNA was then hybridized to the Rat Gene 1.0 ST microarrays containing more than 27,000 transcripts (Affymetrix, Santa Clara, CA, USA). Hybridized chips were scanned on Affymetrix Scanner 3000. CEL files containing raw data were then pre-processed and analyzed with Partek Genomic Suite 6.5 beta software (Partek Inc, St. Louis, MO) using RMA, GC-content adjusted algorithm. Lists of differentially expressed genes treatment were generated using following cut off criteria: signal ratio Control/Treatment >1.20 or <0.83, mean difference for un-logged signals between control and treatment >10, t-test p-values<0.05. CEL files from this study have been deposited with the NCBI gene expression and hybridization array data repository (GEO, http://www.ncbi.nlm.nih.gov/geo, #GSE# GSE24666 and can be also accessed through www.skinner.wsu.edu, and is MIAME compliant. For genes annotation, Affymetrix annotation file RaGene1_0stv1.na30.rn4.transcript.csv was used unless otherwise specified. Generation of affected KEGG pathways (Kyoto Encyclopedia for Genes and Genome, Kyoto University, Japan) used Pathway-Express, a web-based tool freely available as part of the Onto-Tools (http://vortex.cs.wayne.edu) [42]. Previous studies have demonstrated that microarray data are validated with quantitative PCR data [38], [43]. Due to the presence of 11 different oligonucleotide sets for each specific gene being used on the microarray versus only a single primer set for a gene in a quantitative PCR, the microarray is more effective at eliminating false positive or negative data and provides a more robust quantification of changes in gene expression.

## Supporting Information

Table S1 Genes differentially expressed in F-E13 with at least 2 Treatments.
(PDF)

Table S2 (S2A) Differential regulation of female E13 cell culture transcriptome by rat Sry expression construct. (S2B) Differential regulation of female E13 cell culture transcriptome by Tcf21 expression construct. (S2C) Differential regulation of female E13 cell culture transcriptome by Tcf12 expression construct. (S2D) Differential regulation of female E13 cell culture transcriptome by Tcf21 plus Tcf12 expression construct.
(PDF)

Table S3 List of primers used in the present study.
(PDF)
