# Supplementary material for: Basic Helix-Loop-Helix Transcription Factor TCF21 Is a Downstream Target of the Male Sex Determining Gene SRY
Source: PLoS One. 2011 May 17;6(5):e19935. doi: 10.1371/journal.pone.0019935 (PMC3101584; doi:10.1371/journal.pone.0019935)
Supplement: Table S1 — Genes differentially expressed in F-E13 with at least 2 Treatments. (PDF) [file pone.0019935.s001.pdf]

Supplemental Table S1

Genes differentially expressed in female E13 cell culture with at least 2 Treatments

Gene Symbol	Con	rSRY	Tcf21	Tcf21 + Tcf12	Tcf12	GeneBank_ RefSeq	ProbeSet ID	GeneTitle	Diffrentially Expressed in the Following Treatments			
Fbxl2	66	54	62	57	54	ENSRNOT00 000033126	10920643	F-box and leucine-rich repeat protein 2	rSRY			Tcf21+Tcf12
Lgals3bp	164	240	182	178	202	NM_139096	10749495	lectin, galactoside-binding, soluble, 3 binding protein	rSRY			Tcf21+Tcf12
Mgp	333	430	334	422	367	NM_012862	10866512	matrix Gla protein	rSRY		Tcf12	
RGD130 6151	74	61	68	62	61	NM_001108 652	10859282	similar to hypothetical protein DKFZp761D0211	rSRY			Tcf21+Tcf12
RGD130 9362	58	78	68	70	78	BC098065	10801973	similar to interferon-inducible GTPase	rSRY			Tcf21+Tcf12
	56	42	42	40	43	ENSRNOT00 000049785	10798501		rSRY	Tcf21	Tcf12	Tcf21+Tcf12
	24	39	33	36	42	---	10859772		rSRY		Tcf12	Tcf21+Tcf12
	79	62	69	59	60	---	10830489		rSRY		Tcf12	Tcf21+Tcf12
	38	58	52	48	52	---	10722425		rSRY	Tcf21		Tcf21+Tcf12
	366	624	455	550	371	---	10834602		rSRY		Tcf12	
