# Supplementary material for: Basic Helix-Loop-Helix Transcription Factor TCF21 Is a Downstream Target of the Male Sex Determining Gene SRY
Source: PLoS One. 2011 May 17;6(5):e19935. doi: 10.1371/journal.pone.0019935 (PMC3101584; doi:10.1371/journal.pone.0019935)
Supplement: Table S2 — (S2A) Differential regulation of female E13 cell culture transcriptome by rat Sry expression construct. (S2B) Differential regulation of female E13 cell culture transcriptome by Tcf21 expression construct. (S2C) Differential regulation of female E13 cell culture transcriptome by Tcf12 expression construct. (S2D) Differential regulation of female E13 cell culture transcriptome by Tcf21 plus Tcf12 expression construct. (PDF) [file pone.0019935.s002.pdf]

Supplemental Table S2A

Differential regulation of female E13 cell culture transcriptome by rat Sry expression construct.

Gene Symbol	**Con**	**rSRY**	Tcf21	Tcf21 + Tcf12	Tcf12	**Ratio SRY/Con**	**Mean_dif rSRY-Con**	GeneBank #	Affymetrix Probe Set ID	GeneTitle
**Cytoskeleton-ECM**										
Lgals3bp	**164**	**240**	182	202	178	**1.47**	**76.7**	NM_139096	10749495	lectin, galactoside-binding, soluble, 3 binding protein
**Development**										
Lrrc8e	**67**	**44**	50	51	51	**0.65**	**-23.6**	NM_001034139	10759553	leucine rich repeat containing 8 family, member E
Mgp	**333**	**430**	334	367	422	**1.29**	**96.8**	NM_012862	10866512	matrix Gla protein
Nnat	**60**	**48**	53	60	52	**0.79**	**-12.3**	NM_053601	10841637	neuronatin
**Growth Factors+Cyto+Chemokines**										
Ccl2	**63**	**86**	69	68	75	**1.36**	**22.8**	NM_031530	10736697	chemokine (C-C motif) ligand 2
Ccl5	**27**	**42**	29	26	29	**1.55**	**15.1**	NM_031116	10745631	chemokine (C-C motif) ligand 5
Ccl7	**343**	**432**	318	393	375	**1.26**	**88.9**	NM_001007612	10736702	chemokine (C-C motif) ligand 7
Cxcl11	**16**	**29**	18	16	18	**1.82**	**13.0**	NM_182952	10771649	chemokine (C-X-C motif) ligand 11
**Immune Response**										
RT1-A1	**83**	**110**	84	91	89	**1.33**	**27.2**	NM_001008827	10828417	RT1 class Ia, locus A1
RT1-CE1	**107**	**139**	107	116	111	**1.30**	**32.0**	NM_001008832	10831105	RT1 class I, CE1
RT1-CE12	**106**	**139**	109	116	111	**1.32**	**33.7**	NM_001008835	10833944	RT1 class I, CE12
RT1-CE13	**59**	**73**	64	60	62	**1.23**	**13.8**	NM_001008836	10827999	RT1 class I, CE13

Ifi44	**47**	**59**	49	49	52	**1.24**	**11.8**	NM_001107729	10827349	interferon-induced protein 44
Ifit1	**66**	**97**	66	66	77	**1.44**	**30.8**	NM_020096	10714907	interferon-induced protein with tetratricopeptide repeats 1
Ifitm3	**76**	**103**	82	89	82	**1.35**	**26.9**	NM_001136124	10726682	interferon induced transmembrane protein 3
**Metabolism & Transport**										
Slc6a15	**68**	**85**	74	68	71	**1.26**	**17.5**	NM_172321	10895251	solute carrier family 6 (neutral amino acid transporter), member 15
Akr1b8	**1172**	**1452**	1110	1033	1235	**1.23**	**279.3**	NM_173136	10854406	aldo-keto reductase family 1, member B8
Apol9a	**33**	**45**	31	32	39	**1.35**	**12.0**	NM_001025066	10905179	apolipoprotein L 9a
**Proteolysis**										
Psmb9	**30**	**40**	33	29	33	**1.36**	**10.6**	NM_012708	10828357	proteasome (prosome, macropain) subunit, beta type 9 (large multifunctional peptidase 2)
Rnf213	**130**	**161**	143	135	137	**1.24**	**30.8**	XM_001081768	10739982	ring finger protein 213
Rnf213	**60**	**75**	61	61	61	**1.24**	**14.8**	XM_001081768	10740000	ring finger protein 213
Timp3	**99**	**81**	103	104	101	**0.81**	**-18.5**	NM_012886	10901231	TIMP metallopeptidase inhibitor 3
**Receptor (Nuclear import)**										
Snupn	**162**	**130**	147	159	160	**0.81**	**-31.1**	NM_001004270	10910252	snurportin 1

**Signaling**										
Farp2	**172**	**139**	173	156	168	**0.81**	**-33.4**	NM_001108 233	10925609	FERM, RhoGEF and pleckstrin domain protein 2
Gbp2	**82**	**112**	79	84	80	**1.36**	**29.7**	NM_133624	10819523	guanylate binding protein 2
RGD130 9362	**58**	**78**	68	78	70	**1.37**	**20.9**	BC098065	10801973	similar to interferon-inducible GTPase
Smad7	**196**	**162**	190	177	173	**0.83**	**-33.8**	NM_030858	10802734	SMAD family member 7
**Small RNA**										
LOC252 890	**74**	**62**	65	68	71	**0.83**	**-12.8**	NR_002705	10820275	Z39 small nucleolar RNA
**Transcription**										
Fbxl2	**66**	**54**	62	54	57	**0.83**	**-11.7**	ENSRNOT00 000033126	10920643	F-box and leucine-rich repeat protein 2
Fosl1	**79**	**96**	78	72	80	**1.22**	**17.5**	NM_012953	10713045	fos-like antigen 1
RGD156 3091	**16**	**26**	15	14	15	**1.62**	**10.3**	ENSRNOT00 000061066	10860801	similar to OEF2
Zfp110	**126**	**102**	106	116	117	**0.81**	**-24.3**	NM_001024 775	10704223	zinc finger protein 110
Znf124	**39**	**26**	32	32	32	**0.66**	**-13.0**	NM_001108 063	10901083	zinc finger protein 124 (HZF-16)
**Translation & Protein Modification**										
Eef2k	**59**	**48**	62	54	55	**0.82**	**-10.8**	NM_012947	10710494	eukaryotic elongation factor-2 kinase
Oasl	**123**	**203**	144	156	162	**1.65**	**79.7**	NM_001009 681	10762740	2'-5'-oligoadenylate synthetase-like
Oasl2	**42**	**62**	43	43	47	**1.47**	**19.4**	NM_001009 682	10762747	2'-5' oligoadenylate synthetase-like 2
Cwc22	**162**	**132**	160	167	156	**0.82**	**-29.5**	ENSRNOT00	10846661	CWC22 spliceosome-

								000017043		associated protein homolog (S. cerevisiae)
Parp12	**123**	**158**	116	119	130	**1.29**	**35.1**	NM_001108623	10862014	poly (ADP-ribose) polymerase family, member 12
Parp14	**91**	**123**	90	89	103	**1.35**	**31.7**	ENSRNOT00000051838	10751469	poly (ADP-ribose) polymerase family, member 14
**Miscellaneous & Unknown**										
Fam49a	**75**	**60**	64	64	68	**0.80**	**-14.9**	NM_001106718	10883636	family with sequence similarity 49, member A
LOC680286	**72**	**87**	75	69	75	**1.22**	**15.8**	ENSRNOT00000017292	10747177	hypothetical protein LOC680286
Depdc6	**91**	**67**	82	87	86	**0.74**	**-24.1**	ENSRNOT00000005722	10896551	DEP domain containing 6
RGD1306151	**74**	**61**	68	61	62	**0.81**	**-13.7**	NM_001108652	10859282	similar to hypothetical protein DKFZp761D0211
RGD1561161	**77**	**62**	66	74	72	**0.80**	**-15.2**	NM_001134510	10813112	similar to BC067074 protein
Rsrc1	**346**	**426**	338	385	371	**1.23**	**80.2**	NM_001014172	10815795	arginine/serine-rich coiled-coil 1
Tmem116	**49**	**38**	43	46	45	**0.79**	**-10.2**	NM_001159625	10762108	transmembrane protein 116
**ESTs**										
	**56**	**42**	42	43	40	**0.75**	**-14.2**	ENSRNOT00000049785	10798501	
	**88**	**133**	94	89	110	**1.50**	**44.5**	ENSRNOT00000053929	10839872	
	**248**	**192**	230	273	214	**0.78**	**-55.7**	---	10926095	
LOC303	**47**	**59**	47	51	50	**1.25**	**12.0**	XR_006768	10746137	hypothetical LOC303431

431										
	**24**	**39**	33	42	36	**1.60**	**14.7**	---	10859772	
	**79**	**62**	69	60	59	**0.79**	**-16.3**	---	10830489	
	**38**	**58**	52	52	48	**1.51**	**19.5**	---	10722425	
	**366**	**624**	455	371	550	**1.72**	**258.1**	---	10834602	
	**21**	**31**	26	25	25	**1.50**	**10.4**	---	10886858	
	**39**	**51**	43	37	35	**1.30**	**11.8**	NM_001111127	10798473	
	**137**	**167**	140	142	149	**1.21**	**29.2**	---	10796226	
	**65**	**45**	57	65	69	**0.68**	**-20.8**	ENSRNOT00000050135	10837097	
	**54**	**65**	55	58	64	**1.21**	**11.0**	---	10902413	
	**45**	**59**	47	44	39	**1.31**	**14.3**	---	10858205	
	**49**	**62**	53	55	46	**1.25**	**12.4**	---	10824719	
	**195**	**160**	174	177	192	**0.82**	**-35.0**	---	10802708	
	**46**	**61**	46	44	45	**1.31**	**14.8**	ENSRNOT00000053250	10766287	
	**41**	**30**	42	32	38	**0.72**	**-11.5**	---	10798547	
	**2057**	**2676**	2074	1798	2264	**1.31**	**618.3**	---	10827450	
	**61**	**74**	66	64	65	**1.22**	**13.3**	---	10827061	
	**61**	**45**	55	47	57	**0.74**	**-15.7**	---	10721218	
	**127**	**104**	126	125	123	**0.82**	**-23.1**	---	10861395	

Female-E13: rSRY vs Control, p<0.05, Fold Change>1.2 , mean_dif>10 - 68 genes (up-regulated 43)

Supplemental Table S2B

Differential regulation of female E13 cell culture transcriptome by Tcf21 expression construct.

Gene Symbol	**Con**	rSRY	**Tcf21**	Tcf21 + Tcf12	Tcf12	**Ratio Tcf21/ Con**	**Mean_ dif Tcf21- Con**	GeneBank #	Affymetrix Probe Set ID	GeneTitle
**Apoptosis**										
Bcl2l11	**65**	57	**54**	60	65	**0.83**	**-10.6**	NM_171988	10839655	BCL2-like 11 (apoptosis facilitator)
Tnfrsf1b	**78**	85	**95**	91	86	**1.21**	**16.4**	NM_130426	10881424	tumor necrosis factor receptor superfamily, member 1b
**Cytoskeleton-ECM**										
Odf3l1	**77**	75	**95**	95	89	**1.25**	**18.9**	NM_001108151	10917717	outer dense fiber of sperm tails 3-like 1
Clec2d	**243**	248	**294**	289	229	**1.22**	**51.3**	NM_130402	10859080	C-type lectin domain family 2, member d
Tnnt2	**537**	522	**880**	914	655	**1.64**	**342.3**	NM_012676	10764244	troponin T type 2 (cardiac)
**Development**										
Fign	**55**	53	**68**	59	60	**1.23**	**13.5**	NM_001106484	10845743	fidgetin
LOC681994	**141**	137	**113**	123	132	**0.80**	**-27.7**	ENSRNOT00000051588	10812390	similar to developmental endothelial locus-1 isoform b
Sema3e	**95**	97	**125**	119	101	**1.32**	**29.9**	NM_001106579	10860457	sema domain, immunoglobulin domain (Ig), short basic domain, secreted, (semaphorin) 3E

**Golgi Apparatus**										
Mgat4a	**25**	28	**37**	29	28	**1.49**	**12.1**	NM_001160155	10927472	mannosyl (alpha-1,3-)-glycoprotein beta-1,4-N-acetylglucosaminyltransferase, isozyme A
**Growth Factors**										
Lif	**92**	87	**122**	104	104	**1.33**	**30.3**	NM_022196	10773853	leukemia inhibitory factor (cholinergic differentiation factor)
**Immune Response**										
Cfh	**171**	185	**129**	160	164	**0.76**	**-41.8**	NM_130409	10768269	complement factor H
LOC685840	**64**	70	**50**	55	50	**0.77**	**-14.3**	ENSRNOT00000035328	10720237	similar to interferon-lambda2
**Metabolism & Transport**										
RGD1561777	**29**	36	**39**	37	36	**1.36**	**10.0**	ENSRNOT00000035047	10833617	similar to Na+ dependent glucose transporter 1
Ccrn4l	**72**	73	**98**	82	72	**1.37**	**26.2**	NM_138526	10815281	CCR4 carbon catabolite repression 4-like (S. cerevisiae)
Efcab7	**87**	78	**72**	84	79	**0.83**	**-15.2**	ENSRNOT00000013089	10870163	EF-hand calcium binding domain 7
Stc2	**245**	267	**293**	276	265	**1.20**	**48.7**	NM_022230	10732644	stanniocalcin 2
Cutc	**60**	70	**75**	64	66	**1.23**	**14.4**	NM_001108525	10730244	cutC copper transporter homolog (E.coli)
Pxdn	**174**	157	**130**	125	157	**0.74**	**-43.9**	ENSRNOT00000060139	10889475	peroxidasin homolog (Drosophila)
**Proteolysis**										

Rnf213	**87**	101	**113**	95	104	**1.29**	**26.1**	XM_001081768	10739998	ring finger protein 213
**Signaling**										
Mrgprb4	**108**	91	**89**	110	96	**0.82**	**-18.9**	ENSRNOT00000045792	10722218	MAS-related GPR, member B4
**Transcription & Signaling Modifications**										
Ccdc46	**66**	56	**54**	63	61	**0.83**	**-11.3**	NM_001105849	10739282	coiled-coil domain containing 46
Neil3	**54**	51	**43**	46	45	**0.80**	**-11.0**	ENSRNOT00000015623	10788059	nei endonuclease VIII-like 3 (E. coli)
Tcf21	**17**	20	**34**	31	18	**2.01**	**17.5**	NM_001032397	10702306	transcription factor 21
Tspyl4	**59**	65	**71**	64	59	**1.21**	**12.5**	NM_001012075	10830285	TSPY-like 4
Zkscan3	**247**	232	**206**	238	254	**0.83**	**-40.6**	NM_001012053	10795335	zinc finger with KRAB and SCAN domains 3
**Miscellaneous & Unknown**										
Fam133b	**169**	146	**135**	156	142	**0.80**	**-33.1**	BC094528	10936823	family with sequence similarity 133, member B
RGD1560017	**95**	100	**79**	81	104	**0.83**	**-16.0**	XM_342480	10847963	similar to Ac2-210
**EST's**										
RGD1311249	**151**	147	**183**	156	163	**1.21**	**32.6**	BC087107	10876281	similar to RIKEN cDNA B230312A22
	**56**	42	**42**	43	40	**0.75**	**-14.0**	ENSRNOT00000049785	10798501	
	**132**	138	**99**	126	120	**0.75**	**-32.9**	NC_001665	10930612	
	**38**	58	**52**	52	48	**1.37**	**14.1**	---	10722425	
	**29**	43	**46**	48	42	**1.59**	**17.2**	---	10939931	
	**79**	88	**64**	82	71	**0.80**	**-15.2**	---	10714056	
	**90**	85	**73**	83	81	**0.81**	**-16.7**	---	10795286	
	**157**	138	**129**	137	138	**0.82**	**-28.2**	---	10901103	
	**2832**	2824	**2304**	2773	2673	**0.82**	**-528.0**	BC090353	10728028	
	**54**	57	**69**	62	66	**1.28**	**15.5**	---	10798255	
	**41**	49	**52**	45	45	**1.27**	**11.4**	---	10785255	
	**56**	61	**71**	59	56	**1.27**	**15.1**	---	10810150	
	**58**	54	**48**	57	57	**0.83**	**-10.2**	---	10723231	

	**52**	49	**42**	46	51	**0.79**	**-10.4**	---	10788549	
	**120**	123	**98**	129	106	**0.82**	**-22.0**	---	10878432	
	**65**	69	**50**	65	54	**0.78**	**-14.6**	---	10714844	
	**868**	904	**1072**	1020	937	**1.23**	**204.2**	---	10713604	
	**206**	193	**168**	169	211	**0.82**	**-37.3**	---	10721700	

Female-E13: Tcf21 vs Control, p<0.05, Fold Change>1.2 , mean_difference>10 - 45 genes (up-regulated 22)

Supplemental Table S2C

Differential regulation of female E13 cell culture transcriptome by Tcf12 expression construct

Gene Symbol	**Con**	rSRY	Tcf21	Tcf21 + Tcf12	**Tcf12**	**Ratio Tcf12/ Con**	**Mean _dif Tcf12- Con**	GeneBank #	Affymetrix Probe Set ID	GeneTitle
**Cell Cycle**										
Cnnm2	**297**	327	324	356	**364**	**1.23**	**66.9**	NM_001011942	10715889	cyclin M2
**Development**										
Mgp	**333**	430	334	367	**422**	**1.27**	**88.2**	NM_012862	10866512	matrix Gla protein
**Growth Factors+Cyto+Chemokines**										
**Angpt1**	**46**	**52**	**49**	**53**	**57**	**1.24**	**11.0**	**NM_053546**	**10903529**	**angiopoietin 1**
**Immune Response**										
LOC685840	**64**	70	50	55	**50**	**0.78**	**-14.1**	ENSRNOT00000035328	10720237	similar to interferon-lambda2
**Metabolism & Transport**										
Slc46a3	**54**	58	61	65	**67**	**1.23**	**12.8**	NM_001024968	10756411	solute carrier family 46, member 3
**Receptors & Binding Proteins**										
Olr1448	**150**	132	134	137	**118**	**0.79**	**-32.1**	NM_001000019	10733888	olfactory receptor 1448
**Signaling**										
Rhpn2	**36**	36	42	41	**46**	**1.29**	**10.4**	NM_001107505	10706146	rhophilin, Rho GTPase binding protein 2
**Transcription**										
Tcf12	**728**	740	736	3485	**4022**	**5.53**	**3293.9**	NM_013176	10918620	transcription factor 12
**Miscellaneous & Unknown**										
LOC498606	**93**	103	97	104	**114**	**1.22**	**20.4**	NM_001025143	10787538	hypothetical protein LOC498606
RGD1307155	**57**	66	65	61	**69**	**1.20**	**11.7**	BC100622	10732521	similar to CG18661-PA

Trim39	**32**	38	36	36	**42**	**1.33**	**10.4**	NM_213562	10827741	tripartite motif-containing 39
**EST's**										
	**544**	570	456	408	**438**	**0.81**	**-105.8**	ENSRNOT00000053268	10909356	
	**56**	42	42	43	**40**	**0.72**	**-15.8**	ENSRNOT00000049785	10798501	
	**24**	39	33	42	**36**	**1.49**	**11.9**	---	10859772	
	**85**	77	85	78	**65**	**0.76**	**-20.4**	ENSRNOT00000052558	10778080	
	**167**	155	158	190	**139**	**0.83**	**-27.8**	---	10755131	
	**37**	43	41	36	**49**	**1.31**	**11.4**	---	10816000	
	**366**	624	455	371	**550**	**1.50**	**183.9**	---	10834602	
	**52**	57	55	58	**62**	**1.20**	**10.5**	---	10712737	
	**21**	27	25	29	**32**	**1.54**	**11.5**	---	10703398	
	**59**	44	68	56	**34**	**0.57**	**-24.9**	37727288	10760298	
	**75**	81	82	79	**95**	**1.27**	**20.2**	---	10800335	
	**79**	62	69	60	**59**	**0.75**	**-19.7**	---	10830489	
	**422**	375	398	390	**344**	**0.81**	**-78.3**	---	10722718	

Female-E13: Tcf12 vs Control, p<0.05, Fold Change>1.2, mean_difference>10 - 24 genes (up-regulated 15)

Supplemental Table S2D

Differential regulation of female E13 cell culture transcriptome by Tcf21 plus Tcf12 expression construct.

Gene Symbol	**Con**	rSRY	Tcf21	**Tcf21 + Tcf12**	Tcf12	**Ratio (Tcf12 +Tcf12)/Con**	**Mean_dif (Tcf12+ Tcf12)- Con**	GeneBank #	Affymetrix Probe Set ID	GeneTitle
**(Hormone)**										
Cort	**36**	31	33	**23**	40	**0.66**	**-12.8**	ENSRNOT00000030302	10881669	cortistatin
**Cell Cycle**										
Fchsd2	**101**	90	103	**82**	94	**0.82**	**-18.5**	NM_001107539	10709135	FCH and double SH3 domains 2
Nuf2	**374**	352	356	**298**	338	**0.80**	**-76.5**	NM_001012028	10769657	NUF2, NDC80 kinetochore complex component, homolog (S. cerevisiae)
**Cytosceleton-ECM**										
Odf3l1	**77**	75	95	**95**	89	**1.24**	**18.1**	NM_001108151	10917717	outer dense fiber of sperm tails 3-like 1
Lgals3bp	**164**	240	182	**202**	178	**1.23**	**38.5**	NM_139096	10749495	lectin, galactoside-binding, soluble, 3 binding protein
Tnnt2	**537**	522	880	**914**	655	**1.70**	**376.7**	NM_012676	10764244	troponin T type 2 (cardiac)
**Development**										
Eid3	**134**	132	138	**109**	129	**0.82**	**-25.0**	NM_001044304	10901436	EP300 interacting inhibitor of differentiation 3
Nav2	**118**	104	119	**98**	109	**0.82**	**-20.9**	ENSRNOT00000046529	10707177	neuron navigator 2

Sema3e	**95**	97	125	**119**	101	**1.26**	**24.2**	NM_001106579	10860457	sema domain, immunoglobulin domain (Ig), short basic domain, secreted, (semaphorin) 3E
Sv2c	**78**	66	76	**59**	68	**0.75**	**-19.1**	NM_031593	10820613	synaptic vesicle glycoprotein 2c
Xtp3tpa	**50**	51	48	**39**	58	**0.78**	**-10.9**	NM_138892	10725989	XTP3-transactivated protein A
**DNA Repair**										
Gins3	**100**	100	91	**81**	95	**0.81**	**-18.4**	NM_001107408	10809122	GINS complex subunit 3 (Psf3 homolog)
Rad54l	**72**	70	67	**59**	63	**0.82**	**-12.8**	NM_001134960	10871151	RAD54 like (S. cerevisiae)
**Immune Response**										
Cd200	**417**	415	479	**513**	436	**1.23**	**96.1**	NM_031518	10751091	Cd200 molecule
Spa17	**94**	89	85	**78**	87	**0.83**	**-16.3**	NM_053482	10916232	sperm autoantigenic protein 17
**Metabolism**										
Lcp1	**234**	219	223	**195**	203	**0.83**	**-39.5**	NM_001012044	10781496	lymphocyte cytosolic protein 1
Pxdn	**174**	157	130	**125**	157	**0.72**	**-49.0**	ENSRNOT00000060139	10889475	peroxidasin homolog (Drosophila)
Slc46a3	**54**	58	61	**65**	67	**1.22**	**11.6**	NM_001024968	10756411	solute carrier family 46, member 3
**Proteolysis**										
LOC286960	**218**	237	256	**279**	240	**1.28**	**61.0**	NM_173301	10862172	preprotrypsinogen IV
**Receptors & Binding Proteins**										

Wbp4	**74**	62	66	**57**	78	**0.77**	**-17.3**	NM_053766	10785468	WW domain binding protein 4 (formin binding protein 21)
**Signaling**										
Pkmyt1	**54**	50	49	**41**	47	**0.76**	**-12.9**	NM_001105766	10731970	protein kinase, membrane associated tyrosine/threonine 1
Prkar2b	**117**	109	112	**98**	108	**0.83**	**-19.6**	NM_001030020	10889590	protein kinase, cAMP dependent regulatory, type II beta
RGD1309362	**58**	78	68	**78**	70	**1.35**	**20.1**	BC098065	10801973	similar to interferon-inducible GTPase
Rgs16	**91**	102	103	**109**	107	**1.20**	**18.2**	NM_001077589	10764773	regulator of G-protein signaling 16
Srpx2	**226**	256	252	**272**	239	**1.20**	**45.2**	NM_001108243	10934865	sushi-repeat-containing protein, X-linked 2
**Transcription**										
Ccdc59	**215**	193	196	**178**	184	**0.83**	**-36.2**	NM_001108090	10895268	coiled-coil domain containing 59
Dnajc30	**100**	87	87	**78**	85	**0.78**	**-22.1**	NM_001109024	10761225	DnaJ (Hsp40) homolog, subfamily C, member 30
Fbxl2	**66**	54	62	**54**	57	**0.81**	**-12.5**	ENSRNOT00000033126	10920643	F-box and leucine-rich repeat protein 2
**Tcf12**	**728**	**740**	**736**	**3485**	**4022**	**4.79**	**2757**	**NM_013176**	**10918620**	**transcription factor 12**

Tcf21	**17**	20	34	**31**	18	**1.85**	**14.4**	NM_001032397	10702306	transcription factor 21
Zfhx2	**35**	40	37	**46**	33	**1.32**	**11.3**	NM_001098803	10783755	zinc finger homeobox 2
Zfp365	**78**	69	68	**62**	72	**0.79**	**-16.1**	NM_001025145	10829791	zinc finger protein 365
Ddx11	**77**	70	65	**64**	68	**0.83**	**-13.4**	ENSRNOT00000016536	10925839	DEAD/H (Asp-Glu-Ala-Asp/His) box polypeptide 11 (CHL1-like helicase homolog, S. cerevisiae)
**Translation**										
Shq1	**92**	78	89	**76**	87	**0.83**	**-15.7**	NM_001134713	10864446	SHQ1 homolog (S. cerevisiae)
**Miscellaneous & Unknown**										
LOC685634	**137**	137	157	**169**	149	**1.23**	**31.7**	XM_001064606	10872155	hypothetical protein LOC685634
LOC689065	**61**	56	61	**51**	55	**0.83**	**-10.3**	NM_001109521	10727597	hypothetical protein LOC689065
RGD1306151	**74**	61	68	**61**	62	**0.83**	**-12.8**	NM_001108652	10859282	similar to hypothetical protein DKFZp761D0211
RGD1309522	**158**	151	156	**125**	142	**0.79**	**-33.4**	NM_001013997	10781794	similar to hypothetical protein FLJ22624
RGD1359529	**294**	301	327	**360**	333	**1.23**	**65.7**	NM_001014193	10872829	similar to chromosome 1 open reading frame 63
RGD1564140	**70**	64	61	**58**	63	**0.83**	**-12.2**	BC166863	10800592	similar to AW554918 protein
Robld3	**43**	45	41	**54**	43	**1.25**	**10.8**	NM_001106441	10824344	roadblock domain containing 3
**ESTs**										

LOC689 296	**86**	76	85	**70**	82	**0.81**	**-15.9**	NM_001109531	10890190	similar to expressed sequence C79407
RGD130 4610	**50**	57	56	**63**	58	**1.27**	**13.6**	NM_001107258	10782971	similar to DNA segment, Chr 14, ERATO Doi 436, expressed
RGD156 3680	**76**	66	69	**62**	75	**0.81**	**-14.1**	XM_575206	10838559	similar to CDNA sequence BC052040
	**544**	570	456	**408**	438	**0.75**	**-136**	ENSRNOT0000005 3268	10909356	
	**56**	42	42	**43**	40	**0.76**	**-12.9**	ENSRNOT0000004 9785	10798501	
	**25**	28	31	**37**	34	**1.46**	**11.7**	ENSRNOT0000005 5695	10873875	
	**417**	456	367	**331**	376	**0.79**	**-85.5**	ENSRNOT0000005 3807	10909360	
	**24**	39	33	**42**	36	**1.74**	**18.0**	---	10859772	
	**79**	62	69	**60**	59	**0.76**	**-19.1**	---	10830489	
	**129**	146	138	**94**	95	**0.74**	**-35.2**	---	10718134	
	**44**	39	40	**33**	35	**0.74**	**-11.4**	---	10711257	
	**38**	46	40	**56**	38	**1.46**	**17.5**	---	10756530	
	**38**	58	52	**52**	48	**1.35**	**13.7**	---	10722425	
	**120**	138	131	**147**	129	**1.23**	**27.7**	---	10871771	
	**15**	19	16	**28**	15	**1.92**	**13.3**	---	10704115	
	**29**	43	46	**48**	42	**1.59**	**18.8**	---	10939931	
	**88**	96	104	**116**	99	**1.32**	**28.0**	ENSRNOT0000005 4517	10797013	
	**88**	96	104	**116**	99	**1.32**	**28.0**	ENSRNOT0000005 4517	10932228	
	**17**	16	20	**29**	21	**1.75**	**12.6**	---	10850319	
	**132**	125	144	**163**	138	**1.24**	**31.6**	ENSRNOT0000005 3376	10736240	
	**98**	93	106	**123**	110	**1.25**	**24.2**	---	10834604	
	**69**	58	65	**57**	67	**0.82**	**-12.3**	---	10839060	
	**291**	323	310	**358**	293	**1.23**	**67.2**	---	10767075	
	**225**	239	261	**271**	224	**1.21**	**46.7**	---	10910770	
	**246**	266	290	**295**	263	**1.20**	**49.3**	---	10749714	
	**15**	20	17	**25**	16	**1.64**	**10.2**	---	10854030	
	**57**	64	64	**83**	58	**1.48**	**26.4**	---	10852985	
	**67**	59	57	**49**	60	**0.73**	**-18.3**	---	10781997	
	**80**	82	71	**58**	70	**0.72**	**-21.8**	---	10900122	
	**31**	32	32	**43**	30	**1.39**	**12.0**	---	10886267	

Female-E13: Tcf21+Tcf12 vs Control, p<0.05, Fold Change>1.2 , mean_difference>10 - 71 genes (up-regulated 34)
