# Supplementary material for: Basic Helix-Loop-Helix Transcription Factor TCF21 Is a Downstream Target of the Male Sex Determining Gene SRY
Source: PLoS One. 2011 May 17;6(5):e19935. doi: 10.1371/journal.pone.0019935 (PMC3101584; doi:10.1371/journal.pone.0019935)
Supplement: Table S3 — List of primers used in the present study. (PDF) [file pone.0019935.s003.pdf]

Supplemental Table S3. List of primers used in the present study.

**Primer Name**	**Forward Primer (5'-3')**	**Reverse primer (5'-3')**
Tcf21 expression	CTCCTCCACCTCCTCTCTAAACATG	CAAGGTCAGGATGCGGTGGTTC
Deletion mutant 1	TCTTGAGAATTT*GGTACC*AAGATAGCTAACCGTCA	AGGGAGCCAGTG*CCCGGG*TTTAGAGAGGAGGTGG
Deletion mutant 2	TCTGTCATCCCTGCTGT*GGTACC*AGTCCTTTCCT	AGGGAGCCAGTG*CCCGGG*TTTAGAGAGGAGGTGG
Deletion mutant 3	ACACTGGTGATTAA*GGTACC*TTGGGTCCTCAAGA	AGGGAGCCAGTG*CCCGGG*TTTAGAGAGGAGGTGG
Deletion mutant 4	AGATCTCTGCTAAC*GGTACC*AAATCTTAGAGGCTAG	AGGGAGCCAGTG*CCCGGG*TTTAGAGAGGAGGTGG
Deletion mutant 5	TCTCTGTGTTCTCA*GGTACC*ATTAGTTGGGATTC	AGGGAGCCAGTG*CCCGGG*TTTAGAGAGGAGGTGG
Tcf21p-WT	ATCTCCACACTGGTGATTAACAAATTTTGGGTCCTCAAGA	TCTTGAGGACCCAAAATTTGTTAATCACCAGTGTGGAGAT
Tcf21p-MutA	ATCTCCACACTGGTGATTATAGTATTTTGGGTCCTCAAGA	TCTTGAGGACCCAAAATACTATAATCACCAGTGTGGAGAT
Tcf21p-MutB*	CAGATCTCTGCTATGTAAACAAAATCTTAGAGGC	GCCTCTAAGATTTTGTTTACATAGCAGAGATCTG
Tcf21p-MutC*	CAGATCTCTGCTATGTAAAGAGAATCTTAGAGGC	GCCTCTAAGATTCTCTTTACATAGCAGAGATCTG
Tcf21p-MutD	GCCTCTAAGATTTTGTTTACATAGCAGAGATCTG	CAGATCTCTGCTATGTAAACAAAATCTTAGAGGC
Tcf21p-MutE	CAGATCTCTGCTAACAAAAGAGAATCTTAGAGGC	GCCTCTAAGATTCTCTTTTGTTAGCAGAGATCTG
Tcf21p- forward sequence -1	CTGCCGTATCTTAGCAATCTGGTA	
Tcf21p- forward sequence -2	CATATCTGAGTGTAGCAGAGAGGC	
Tcf21p- forward sequence-3	ATCGGCCCAGTGATCAGCATGTT	
Tcf21p- reverse sequence	GATGACAGAAATATCAAGTCGAGGT	
Tcf21p-ChIP primer for SRY	TCTCCACACTGGTGATTAACAAA	TAATCCAGGCTCAGCTGAGA
Tcf21p-ChIP primer for GATA4	AGGGGTCTAGGAAGTCTGCTG	TTCTGTGTGTTGGGTTTCCA
Tcf21p-ChIP non-specific primer	AGCAGTTCCCTGATACCTTCA	TCGCAGTCATAGGTCCTGTCT
AMH** mRNA	AAAGAAGGTGCCACCCTGAC	GTAATAGGGGTTCCTCCCCA
AMH** mRNA for nesting	AAGAAGGTGCCACCCTGAC	GTCAGAGGTCCCCGCAG
L19 housekeeping	CTGAAGGTCAAAGGGAATGTG	GGACAGAGTCTTGATGATCTC

* MutA was used as a PCR template for generating these constructs. ** primers including exon-intron boundary

Mutation of the binding sites are underlined and the restriction sites are *italicized*.
